# The Antioxidant Role of Hemp Phytocomplex in Cannabis Oil-Based Extracts

**DOI:** 10.3390/ph15091102

**Published:** 2022-09-04

**Authors:** Camillo Morano, Michele Dei Cas, Gabriella Roda, Adalberto Fabbriconi, Eleonora Casagni, Marco Pallavicini, Cristiano Bolchi, Gloria Pallotti, Francesco Romaniello, Pierangela Rovellini

**Affiliations:** 1 Department of Pharmaceutical Sciences, Università degli Studi di Milano, 20133 Milan, Italy; 2 Department of Health Sciences, Università degli Studi di Milano, 20142 Milan, Italy; 3 Farmacia Bandi, 20127 Milan, Italy; 4 INNOVHUB-SSI srl, 20133 Milan, Italy

**Keywords:** cannabis, antioxidants, galenic formulas, olive oil, MCT

## Abstract

The therapeutic use of Cannabis oil extracts is constantly increasing. However, in Italy, they are allowed to be prepared with only a few methods and matrices. With this work, we aimed to assess how the different processes might affect the chemical composition of two different matrices (olive oils and medium chain triglycerides oils - MCT), accounting as variables for both the presence of Cannabis dried apices of the female flower and the adding of tocopherol acetate as an antioxidant. The macerated oils were prepared with four of the methods allowed according to the Italian legislation (Romano-Hazekamp, Cannazza-Citti, SIFAP and Calvi) and analyzed for normal and oxidized tocopherols, oxidized and conjugated fatty acids and volatile carbonyl compounds (VCCs), all using liquid chromatography coupled to UV or PDA detectors. According to our results, neither normal nor oxidized tocopherols are affected by the addition of antioxidants or Cannabis, while the oxidation state (according to the levels of oxidized and conjugated fatty acids) is often altered in either case. The VCCs concentrations, on the other hand, are never notably altered. These results suggest a worthless use of antioxidants in Cannabis macerated oils preparations, while the dried apices of female flowers might have a protective role in maintaining the oil oxidation state.

## 1. Introduction

After decades of legal restrictions and prohibitions, the use of *Cannabis sativa* L. (hereinafter, cannabis) for medical purposes has significantly increased in the past few years. Its clinical benefits include the symptomatic treatment of anorexia, pharmacological-induced nausea, multiple sclerosis, AIDS and many other conditions [1,2,3]. The pharmacological and psychoactive effects of cannabis are mostly attributed to the cannabinoids present in the plant, particularly delta-9 tetrahydrocannabinolic acid (THCA) and cannabidiolic acid (CBDA), which become active after decarboxylation to delta-9 tetrahydrocannabinol (THC) and cannabidiol (CBD) [4]. However, many authors have stipulated how these properties can also be ascribed to or modulated by other components of the plant, such as the terpenes, that contribute to the so-called *entourage effect* [5,6,7,8]. More and more often, cannabis is administered as a macerated oil, as its dosage can be easily adjustable, and its components have an increased bioavailability [9]. Maceration is a technique that involves the infusion of the dried parts of the plant, properly minced to expose a larger surface area in an appropriate liquid medium—in this case, fat oils—for the extraction of the plant’s active components. To prepare these oils, the Italian jurisdiction allows different methods proposed by Società Italiana Farmacisti Preperatori (SIFAP), Romano Hazekamp, Cannazza and Calvi [10,11,12,13]. Pharmacies manufacture these preparations as fit-to-patient drugs, known as galenic formulas. Our work aimed to determine how the preparation and treatment of the sample could affect the oxidation state of some oil-based galenic formulas for the administration of cannabis. The procedures in analysis were Romano-Hazekamp (method A), Cannazza (method B), SIFAP (method C) and Calvi (method D). The oils used for the preparation of the medicaments were olive oils and medium-chain triglyceride oils (MCT). In order to reach our goal with these materials, we characterized and quantified the natural tocopherols and oxidized forms, conjugated fatty acids, oxidized fatty acids, trienes and carbonylic volatile compounds (VCCs) present in the galenic formulas submitted to the study.

## 2. Results and Discussion

### 2.1. Determination of Tocopherols and their Oxidized Forms

**Figure 1:**
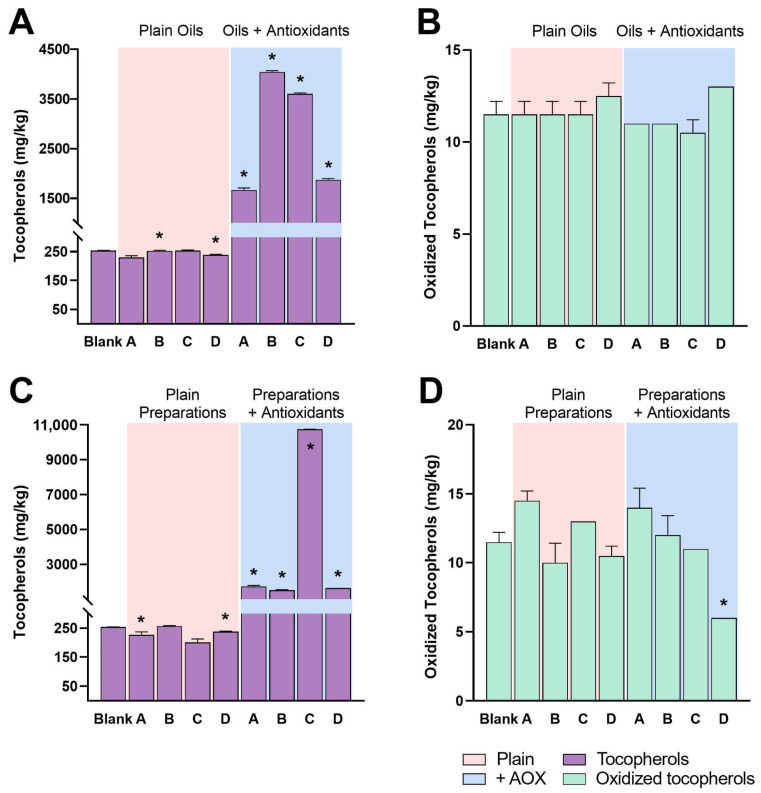
Tocopherols and their oxidized forms in oils and cannabis pharmaceutical preparations and their impact on their concentrations after adding an antioxidant. (**A**) Tocopherols content in oils. (**B**) Oxidized forms of tocopherols in oils. (**C**) Tocopherols content in pharmaceutical preparations. (**D**) Oxidized forms of tocopherols in pharmaceutical preparations. Preparation methods are summarized as method A Hazekamp et al., B Cannazza et al., C SIFAP, and D Calvi et al.; see the main text for details. * refers to significant differences (*p* < 0.05) in concentrations of tocopherols or their oxidized forms against blank.

The concentrations of tocopherols and their oxidized forms are plotted in Figure 1 and data are reported in extenso in Supplementary Tables S1 and S3. **Panels A–B** shows the differences that incur along each method applied to plain and antioxidant-added olive oil. The blank sample displays a concentration of 254 mg/kg of tocopherols and 12 mg/kg of oxidized tocopherols. In face of this finding, methods A and C do not seem to influence the degradation of the tocopherols, while methods B and D provided an 8–10% decrease of the total tocopherols, though this variation falls into the measurement error of the analytical method. No variations were recorded in the concentrations of oxidized tocopherols. However, these same remarks cannot be easily made for the antioxidant-added oils, as the additions of α-tocopherol acetate were not uniform, varying from 1500 to 3000 mg/kg. The trend in concentrations remains basically the same in the pharmaceutical preparations (**Panels C–D**). Results are not graphed for any MCT oil and preparation, as it naturally does not contain tocopherols (Supplementary Tables S2 and S4).

### 2.2. Determination of Oxidized and Conjugated Fatty Acids 

**Figure 2:**
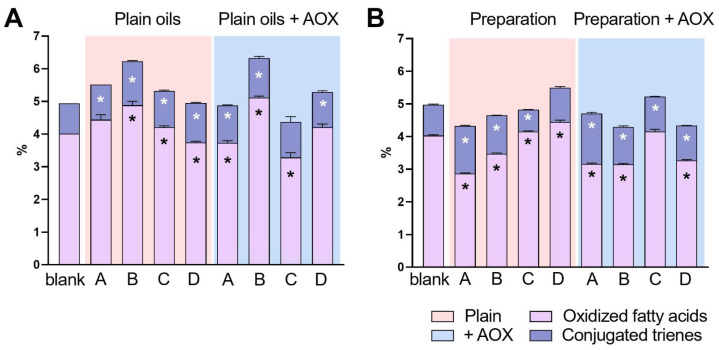
Percentage of oxidation by-products of fatty acids in oils (**A**), the cannabis pharmaceutical preparations (**B**), and the impact on their concentrations after adding an antioxidant. Preparation methods are summarized as method A (Hazekamp et al.), B (Cannazza et al.), C (SIFAP) and D (Calvi et al.); see the main text for details. * refers to significant differences in oxidized fatty acids (black *) and conjugated trienes (white *) against blank (olive oil).

The sample not subjected to any treatment displays 4.01% and 0.93% of oxidized fatty acid and conjugated trienes, respectively. Heat treatments, such as those for the production of oil extracts, should cause an incremental change in these species, and the empirical data are in line with this hypothesis (Figure 2, Supplementary Tables S5 and S7). Method D maintains an approximately unchanged oxidation state (3.75% vs. 4.01%) but increases to 1.20% in triene species. All other methods promoted a general increase in the total oxidation state; mainly, method B produced an increment in oxidized species by about 20% (4.88% vs. 4.01%), and the trienes showed a rise of 44% (1.34% vs. 0.93%). Exploiting methods A and C, and by adding an antioxidant such as α-tocopherol acetate, no increasing trend is observed because the values are within the reproducibility value (for a level of 3.46%, reproducibility value = 1.42%), and the triene values remain stable or slightly higher (Method A 1.09%/ 0.93%; Method C 1.14%/0.93%). Unfortunately, the expected protective effect was not found in the samples treated with methods B and D; in fact, there was an increase in the oxidation state. However, by examining the concentration of quantified antioxidants in detail, it was observed that it was half of that added in the samples mentioned above (Method A and C). Therefore, it is difficult to establish whether the two methods are exceptionally stressful or whether the amount of antioxidants is insufficient to produce a beneficial effect. The preparations (Figure 2B) make the situation extremely complex. The addition of Bedrocan seems to produce a protective effect on the oil. In fact, treatments with methods A, B and C display an equal or even lower value of oxidized fatty acids compared to a blank (2.86%, 3.46% and 4.17% vs. 4.01%).

On the other hand, these data are not reconfirmed if the production process is method D, as there is, in fact, an increase in oxidized fatty acids (4.41%). Samples with α-tocopherol acetate also have generally lower oxidation values than the reference, except for those treated with method C. However, it is difficult to understand if the effect is due to the dried apices of the female flower (as in the previous sample set) or to the α-tocopherol acetate, whose resulting concentration is approximately 1300–1900 mg/kg. Results are not graphed for any MCT oil and preparation, as all analytes were under LOQ in all samples (Supplementary Tables S6 and S8). 

### 2.3. Determination of Volatile Carbonyl Compounds (VCCs)

**Figure 3:**
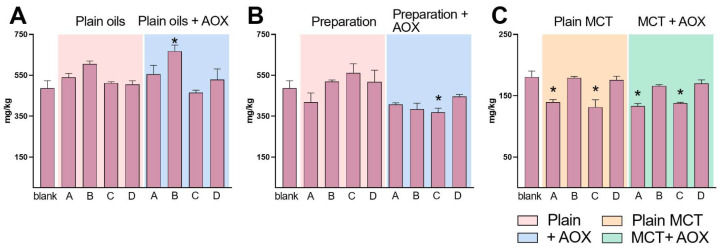
Volatile carbonyl compounds (VCCs) in oils (**A**), cannabis pharmaceutical preparations (**B**), and medium chain triglycerides oils (MCT, (**C**) and the impact on their concentrations after the addition of an antioxidant. Preparation methods are summarized as method A (Hazekamp et al.), B (Cannazza et al.), C (SIFAP) and D (Calvi et al.); see the main text for details. * refers to significant differences in VCCs concentrations against blank.

The last monitored oxidation parameter is the carbonyl compounds (Figure 3). The reference sample shows a wide spectrum of volatile components. From propanal to pentadecanal with a final concentration of 486.6 mg/kg, it is worth noticing that the total aldehydes for extra virgin olive oil within its expired data should be ≤ 200 mg/kg. From our experience [14], the high-temperature treatment raises the overall content of the volatile aldehyde and ketone components from a minimum of 505 mg/kg with method D to a maximum of 605 mg/kg with method B. The addition of α-tocopherol acetate, referring exclusively to these oxidation compounds, does not seem to have a well-defined effect. However, the samples processed with the other methods have higher values in the concentration of volatile components (Supplementary Table S9). MCTs, just like olive oils and olive-based oil extracts, do not identify a real trend. As fewer types of fatty acids exist, the number of volatile components produced is also limited. In detail, the following were identified and quantified: heptanal, octanal, nonanal and decanal. Before the treatments, the MCT records a value of volatile compounds equal to 181 mg/kg made mainly up of C8 (about 120 mg/kg) and C10 (about 47 mg/kg) (Supplementary Table S10). Methods B and D keep this value stable. 

On the contrary, the samples treated with methods A (131 mg/kg) and C (140 mg/kg) showed an even lower content. The same trend is shown for the samples added with antioxidants, which therefore does not seem to demonstrate a great efficacy in reducing the formation of volatile compounds. Even the presence of an active component such as Bedrocan does not generate a real trend. 

## 3. Materials and Methods

### 3.1. Chemicals and Reagents

Materials included refined olive oils and the powder of *Canapa sativa* L. extract. The chemicals 2-propanol, acetone, acetonitrile, hexane, methanol, and water (all analytical grade) were purchased from Sigma-Aldrich (St. Louis, MO, USA). Acetic acid (99.8% purity) and perchloric acid (70–72% purity) were purchased from Honeywell Fluka (Buchs, Germany). Reagents such as 2,4-dinitrophenylhydrazine (reagent grade, 97%) and sodium benzyloxide solution (1.0 M solution in benzyl alcohol) were purchased from Merck (Darmstadt, Germany). Analytical standards (±)-α-tocopherol (≥96% HPLC grade), D-α-tocopherylquinone (≥96% HPLC grade), lauric aldehyde (purity ≥ 95%) were purchased from Sigma-Aldrich (St. Louis, MO, USA); tricaproin and triheptadecanoin (purity ≥ 99%) from Larodan (Solna, Sweden).

### 3.2. Samples

Samples (n. 28) were gathered and analyzed, distributed as follows: n. 2 blanks (olive oil and MCT both at Pharmacopoeia Grade), n. 4 olive oil samples, n. 4 olive oil samples added with antioxidant (tocopherol acetate, 0.2% *w*/*v*), n. 4 olive oil Cannabis preparations (Bedrolite, Bedrocan and Bediol) n. 4 olive oil Cannabis preparations added with antioxidant, n. 4 MCT samples, n. 4 MCT samples treated with antioxidant, n.1 MCT Cannabis (Bedrocan) preparation and n.1 MCT Cannabis (Bedrocan) preparation added with antioxidant. All the samples, with the exception of the blanks and the two MCT preparations, were made with the four different methods in analysis: A— Hazekamp [11], B—Citti/Cannazza [12], C—SIFAP [10] and D—Calvi [13]). The two MCT preparations were made using the SIFAP method (C). All samples were stocked at refrigerated temperatures (5 °C) and kept away from light until analysis.

The preparation methods for the cannabis medicinal oils are mainly based on the maceration of vegetable materials in olive oil at high temperature, at about 100 °C or over (Methods A—Hazekamp et al. [11] and B—Citti/Cannazza et al. [12]). Both of them do not require a preliminary decarboxylation of the vegetal matrix. A preliminary decarboxylation step is performed with Method C—SIFAP [10] or Method D—Calvi et al. [13]. These methods were used by pharmacists based on medical prescriptions to obtain cannabis oils by using either Bedrolite, Bedrocan, or Bediol varieties of medicinal-grade plant material (Table 1). Plain oils were treated as described above, except for the maceration of vegetable materials, which was missed here. Both the cannabis pharmaceutical preparations and plain oils were possibly added with tocopherol acetate (0.2% *w*/*v*) as an antioxidant.

### 3.3. Methods

#### 3.3.1. Determination of Tocopherols and their Oxidized Forms

For the determination of tocopherols and their oxidized forms, 300 ± 10 mg of homogenized oil samples was placed in a 10 mL amber volumetric flask. The samples were made up to volume with acetone and vigorously shaken. After a suitable filtration on a PVDF 0.22 µm filter, the samples were ready for the HPLC-PDA injection. The Thermo Finnigan (San Jose, CA, USA) HPLC P4000 was coupled with an UV 6000LP (Thermo Finnigan, San Jose, CA, USA), and the spectra acquisition range was between 200 and 400 nm. The chromatographic separation was performed on Allsphere C18 column 5 µm, 250 × 4.6 mm (SepaChrom, Rho, MI, Italy) by an isocratic elution with methanol and acetonitrile (1:1) at 1.30 mL/min of flow rate, maintained for 15 minutes; the injection volume was 20 µL. The chromatograms were extracted at 292 nm for the determination and quantification of α, β + γ and δ tocopherol and at 268 nm for the quantification of α-tocopherylquinone, epoxy-α-tocopherolquinone and its isomeric form. The quantification was made through an external standard calibration curve. All the tocopherols’ forms were quantified as α-tocopherol used as an external standard, and oxidized forms were quantified using α-tocopherylquinone as an external standard [15].

The determination of tocopherols and their oxidized forms in cannabis oil extract preparations required a more complex chromatographic separation due to many matrix interferences. The conditions were set as follows: the chromatographic separation was performed on a reversed-phase Luna C18 column 5 µm, 250 × 4.6 mm (Phenomenex, Torrance, CA, USA) by a gradient between (A) water, (B) methanol and (C) acetonitrile starting at 40:30:30 (v:v:v) and increased to 0:50:50 (v:v:v) after 15 minutes for a runtime of 25 minutes. The flow rate was set to 1.5 mL/min.

#### 3.3.2. Determination of Oxidized and Conjugated Fatty Acids 

Oxidized and conjugated fatty acids were determined as benzyl esters derivatives according to [16]. The Nexera Shimadzu (Kyoto, Japan) HPLC was coupled with a SIL-20A autosampler and SPD-20A UV detector. The method is based on the transesterification of fatty acids with a sodium benziloxide solution of 1.0 M. Approximately a 500 ± 10 mg of sample was put in a 25 mL volumetric flask made up to volume with anhydrous hexane. Then, 1 mL of the sample solution was transferred to a test tube and spiked with 1 mL of a mixed solution of tricaproin (200 mg/L) and triheptadecanoin (400 mg/L). The samples were allowed to react for 15 min after the addition of 50 µL of benziloxide solution. The samples were then centrifuged, and the upper layer dried under nitrogen at room temperature; the samples were then reconstituted with 1000 µL 2-propanol, and 20 µL were injected into the already described HPLC-PDA system. The elution was achieved with a linear gradient of 50 minutes from 40% of (A) water and 60% of (B) acetonitrile to 100% of (B) with a flow rate of 1 mL/min. For the quantification, the chromatograms were extracted at 255 nm. All the compounds were quantified, with triheptadecanoin used as an internal calibrator; the results expression is well detailed in [16]. The completion of the transesterification reaction was verified by calculating the ratio between the response factors (RF) of the tricaproin and triheptadecanoin.

#### 3.3.3. Determination of Volatile Carbonyl Compounds (VCCs)

Lipophilic volatile carbonyl compounds (VCCs), such as aldehydes and ketones, could be originated from the autoxidation of fatty acids. The VCCs were derivatized with a 2,4-Dinitrophenylhydrazine solution (0.1% *w*/*v* in acetonitrile, HClO_4_ 0.01 N) and chromatographically separated, identified and quantified using HPLC-PDA. The Thermo Finnigan (San Jose, CA, USA) HPLC P4000 was coupled with an UV 6000LP (Thermo Finnigan, San Jose, CA, USA), and the spectrum acquisition range was between 200 and 600 nm. Portions of oil of about 100 ± 10 mg were placed in test tubes with 50 µL of a lauric aldehyde solution (500 mg/L) as the internal standard. One milliliter of the derivative solution was added, and the reaction was promoted with an ultrasonic bath for 15 min at room temperature. After centrifugation (3000 rpm, 15 min), the upper layer was transferred to an amber vial, and 20 µL were injected into the HPLC system. The chromatographic separation was performed on a reverse-phase C18 column, Allsphere ODS2 (SepaChrom, Rho, MI, Italy), 5 µm 250 × 4.6 mm by a gradient between (A) water, (B) methanol and (C) acetonitrile starting at 40:30:30 (v:v:v) and increased to 0:50:50 (v:v:v) in 60 minutes. The flow rate was set to 0.9 mL/min. The quantification was performed at 360 nm for all the analytes, using lauric aldehyde as internal standard [14].

### 3.4. Statistical Analysis

Visualization of the results and univariate statistical analysis were attained using GraphPad Prism 9.0 (GraphPad Software, Inc, La Jolla, CA, USA). For comparison among groups, a *t*-test was performed. In all tests, *p* < 0.05 was considered statistically significant.

## 4. Conclusions

With our work, we evaluated whether four of the most used methods for the preparation of galenic cannabis macerated oils, namely Romano-Hazekamp, Cannazza, SIFAP and Calvi, could affect the composition and properties of olive oil or MCT, the preferred matrices for oral administration, and the role of Cannabis dried apices of female flower in above-mentioned changes, with and without the addition of α-tocopherol acetate as an antioxidant. Tocopherols and oxidized tocopherols in olive oil were not particularly altered with any of the methods, while the oxidation state, determined as oxidized and conjugated fatty acids in the same matrix, seemed to be reduced with the addition of the cannabis only. The addition of an antioxidant, on the other hand, did not seem to produce any appraisable effect. This could suggest that the hemp phytocomplex plays a protective role from oxidation in its oil extracts. Moreover, the cannabis variety, as expected, did not influence any of the studied properties in the macerated oils.

## Figures and Tables

**Table 1:** Cannabinoids concentrations in oil extracts depending on the preparation methods and the cannabis varieties.

Cannabis Varieties	Preparation Methods	THC tot (% *w*/*w*)	CBD tot (% *w*/*w*)
Bediol	A. Romano-Hazekamp	0.48	0.65
Bedrocan	B. Citti-Cannazza	1.49	-
Bedrocan	C. Sifap	1.52	-
Bedrolite	C. Sifap	-	0.60
Bedrocan	D. Calvi	1.23	-
Bediol	D. Calvi	0.31	0.53

## Data Availability

Not applicable.

## Supplementary Materials

The following supporting information can be downloaded at: https://www.mdpi.com/article/10.3390/ph15091102/s1, Table S1. Tocopherols (mg/kg) in olive oils and cannabis pharmaceutical preparations and the impact on their concentrations after the addition of tocopherol acetate as an antioxidant (AOX). Table S2. Tocopherols (mg/kg) in MCT oils and cannabis pharmaceutical preparations and the impact on their concentrations after the addition of tocopherol acetate as an antioxidant (AOX). Table S3. Oxidized tocopherols (mg/kg) in olive oils and cannabis pharmaceutical preparations and the impact on their concentrations after the addition of tocopherol acetate as an antioxidant (AOX). Table S4. Oxidized tocopherols (mg/kg) in MCT oils and cannabis pharmaceutical preparations and the impact on their concentrations after the addition of tocopherol acetate as an antioxidant (AOX). Table S5. Percentage of oxidized fatty acids in olive oils and cannabis pharmaceutical preparations and the impact on their concentrations after the addition of tocopherol acetate as an antioxidant (AOX). Table S6. Percentage of oxidized fatty acids in MCT oils and cannabis pharmaceutical preparations and the impact on their concentrations after the addition of tocopherol acetate as an antioxidant (AOX). Table S7. Percentage of trienes conjugated fatty acids in olive oils and cannabis pharmaceutical preparations and the impact on their concentrations after the addition of tocopherol acetate as an antioxidant (AOX). Table S8. Percentage of conjugated fatty acids trienes in MCT oils and cannabis pharmaceutical preparations and the impact on their concentrations after the addition of tocopherol acetate as an antioxidant (AOX). Table S9. Volatile carbonyl compounds (VCCs) in olive oils and cannabis pharmaceutical preparations and the impact on their concentrations after the addition of tocopherol acetate as an antioxidant (AOX). Table S10. Volatile carbonyl compounds (VCCs) in MCT oils and cannabis pharmaceutical preparations and the impact on their concentrations after the addition of tocopherol acetate as an antioxidant (AOX).
