# Supplementary material for: The Antioxidant Role of Hemp Phytocomplex in Cannabis Oil-Based Extracts
Source: Pharmaceuticals (Basel). 2022 Sep 4;15(9):1102. doi: 10.3390/ph15091102 (PMC9503170; doi:10.3390/ph15091102)
Supplement: Supplementary file 1 [file pharmaceuticals-15-01102-s001.zip › pharmaceuticals-1884668-supplementary.pdf]

pharmaceuticals

Article

The Antioxidant Role of Hemp Phytocomplex in Cannabis Oil-Based Extracts

Camillo Morano [1], Michele Dei Cas [2,*], Gabriella Roda [1,*], Adalberto Fabbriconi [3], Eleonora Casagni [1], Marco Pallavicini [1], Cristiano Bolchi [1], Gloria Pallotti [4], Francesco Romaniello [4] and Pierangela Rovellini [4]

1 Department of Pharmaceutical Sciences, Università degli Studi di Milano, Milan, 20133, Italy
2 Department of Health Sciences, Università degli Studi di Milano, Milan, 20142, Italy
3 Farmacia Bandi, Milan, 20127, Italy
4 INNOVHUB-SSI srl, Milan, 20133, Italy
* Correspondence: michele.deicas@unimi.it (M.D.C.); gabriella.roda@unimi.it (G.R.)

Supplementary Materials

Table S1. Tocopherols (mg/kg) in olive oils and cannabis pharmaceutical preparations and the impact on their concentrations after the addition of tocopherol acetate as antioxidant (AOX).

Table S2. Tocopherols (mg/kg) in MCT oils and cannabis pharmaceutical preparations and the impact on their concentrations after the addition of tocopherol acetate as antioxidant (AOX).

Table S3. Oxidized tocopherols (mg/kg) in olive oils and cannabis pharmaceutical preparations and the impact on their concentrations after the addition of tocopherol acetate as antioxidant (AOX).

Table S4. Oxidized tocopherols (mg/kg) in MCT oils and cannabis pharmaceutical preparations and the impact on their concentrations after the addition of tocopherol acetate as antioxidant (AOX).

Table S5. Percentage of oxidized fatty acids in olive oils and cannabis pharmaceutical preparations and the impact on their concentrations after the addition of tocopherol acetate as antioxidant (AOX).

Table S6. Percentage of oxidized fatty acids in MCT oils and cannabis pharmaceutical preparations and the impact on their concentrations after the addition of tocopherol acetate as antioxidant (AOX).

Table S7. Percentage of trienes conjugated fatty acids in olive oils and cannabis pharmaceutical preparations and the impact on their concentrations after the addition of tocopherol acetate as antioxidant (AOX).

Table S8. Percentage of trienes conjugated fatty acids in MCT oils and cannabis pharmaceutical preparations and the impact on their concentrations after the addition of tocopherol acetate as antioxidant (AOX).

Table S9. Volatile carbonyl compounds (VCCs) in olive oils and cannabis pharmaceutical preparations and the impact on their concentrations after the addition of tocopherol acetate as antioxidant (AOX).

Table S10. Volatile carbonyl compounds (VCCs) in MCT oils and cannabis pharmaceutical preparations and the impact on their concentrations after the addition of tocopherol acetate as antioxidant (AOX).

Table S1. Tocopherols (mg/kg) in olive oils and cannabis pharmaceutical preparations and the impact on their concentrations after the addition of tocopherol acetate as antioxidant (AOX).

		plain oils				oils + AOX				plain preparations				preparations + AOX			
	**BLANK**	**A**	**B**	**C**	**D**	**A**	**B**	**C**	**D**	**A**	**B**	**C**	**D**	**A**	**B**	**C**	**D**
δ-Tocopherol	nd	nd	nd	nd	nd	nd	nd	nd	nd	nd	nd	nd	nd	nd	nd	nd	nd
β+γ Tocopherol	22	21	21	20	22	21	22	22	22	14	19	32	22	21	30	19	16
α-Tocopherol	231	232	209	232	217	237	206	246	232	186	208	224	216	194	223	233	208
α-Tocopherol acetate	nd	nd	nd	nd	nd	3345	1439	3772	1618	nd	nd	nd	nd	10535	1494	1273	1427

Nd= not detectable that should be intended as inferior to 1 mg/kg. Preparation methods are summarized as: method **A** Hazekamp, **B** Cannazza, **C** SIFAP and **D** Calvi; see the main text for details.

Table S2. Tocopherols (mg/kg) in MCT oils and cannabis pharmaceutical preparations and the impact on their concentrations after the addition of tocopherol acetate as antioxidant (AOX).

		plain MCT				MCT + AOX				plain preparations in MCT				Preparations in MCT + AOX			
	**BLANK**	**A**	**B**	**C**	**D**	**A**	**B**	**C**	**D**	**A**	**B**	**C**	**D**	**A**	**B**	**C**	**D**
δ-Tocopherol	nd	nd	nd	nd	nd	nd	nd	nd	nd	-	-	nd	-	-	-	-	-
β+γ Tocopherol	nd	nd	nd	nd	nd	nd	nd	nd	nd	-	-	nd	-	-	-	21	-
α-Tocopherol	nd	nd	nd	nd	nd	nd	nd	nd	nd	-	-	nd	-	-	-	194	-
α-Tocopherol acetate	nd	nd	nd	nd	nd	nd	nd	nd	nd	-	-	nd	-	-	-	10535	-

Nd= not detectable that should be intended as inferior to 1 mg/kg. Preparation methods are summarized as: method **A** Hazekamp, **B** Cannazza, **C** SIFAP and **D** Calvi; see the main text for details.

Table S3. Oxidized tocopherols (mg/kg) in olive oils and cannabis pharmaceutical preparations and the impact on their concentrations after the addition of tocopherol acetate as antioxidant (AOX).

		plain oils				oils + AOX				plain preparations				preparations + AOX			
	**BLANK**	**A**	**B**	**C**	**D**	**A**	**B**	**C**	**D**	**A**	**B**	**C**	**D**	**A**	**B**	**C**	**D**
Epoxy α-Tocopherol	2	4	3	4	4	3	3	3	4	1	1	2	1	1	1	1	1
Epoxy 1 α-Tocopherol	nd	nd	nd	nd	nd	nd	nd	nd	nd	nd	nd	nd	nd	nd	nd	nd	nd
α-Tocopherol quinone	9	8	8	8	9	7	8	7	9	12	14	9	9	10	13	10	5

Nd= not detectable that should be intended as inferior to 1 mg/kg. Preparation methods are summarized as: method **A** Hazekamp, **B** Cannazza, **C** SIFAP and **D** Calvi; see the main text for details.

Table S4. Oxidized tocopherols (mg/kg) in MCT oils and cannabis pharmaceutical preparations and the impact on their concentrations after the addition of tocopherol acetate as antioxidant (AOX).

		plain MCT				MCT + AOX				plain preparations in MCT				Preparations in MCT + AOX			
	**BLANK**	**A**	**B**	**C**	**D**	**A**	**B**	**C**	**D**	**A**	**B**	**C**	**D**	**A**	**B**	**C**	**D**
Epoxy α-Tocopherol	nd	nd	nd	nd	nd	nd	nd	nd	nd	-	-	nd	-	-	-	nd	-
Epoxy 1 α-Tocopherol	nd	nd	nd	nd	nd	nd	nd	nd	nd	-	-	nd	-	-	-	nd	-
α-Tocopherol quinone	nd	nd	nd	nd	nd	nd	nd	nd	nd	-	-	nd	-	-	-	nd	-

Nd= not detectable that should be intended as inferior to 1 mg/kg. Preparation methods are summarized as: method **A** Hazekamp, **B** Cannazza, **C** SIFAP and **D** Calvi; see the main text for details.

Table S5. Percentage of oxidized fatty acids in olive oils and cannabis pharmaceutical preparations and the impact on their concentrations after the addition of tocopherol acetate as antioxidant (AOX).

		plain oils				oils + AOX				plain preparations				preparations + AOX			
	**BLANK**	**A**	**B**	**C**	**D**	**A**	**B**	**C**	**D**	**A**	**B**	**C**	**D**	**A**	**B**	**C**	**D**
OX 230	0.42	0.52	0.64	0.53	0.46	0.38	0.63	0.39	0.46	0.73	0.95	0.44	0.57	0.67	0.59	0.47	0.63
OX 270	3.59	3.92	4.24	3.68	3.29	3.36	4.49	4.49	3.75	2.13	2.51	3.74	3.84	2.46	2.54	3.73	2.62

Nd= not detectable that should be intended as inferior to 0.001%. Preparation methods are summarized as: method **A** Hazekamp, **B** Cannazza, **C** SIFAP and **D** Calvi; see the main text for details.OX 230= sum of hydroxyl, hydroperoxy, epidioxy and cheto C18:1 fatty acids

OX 230= sum of hydroxyl-, hydroperoxyl-, epidioxy- and cheto- oleic (C18:1) fatty acids
OX 270= sum of cheto-linoleic (C18:2) and linolenic (C18:3) fatty acids

Table S6. Percentage of oxidized fatty acids in MCT oils and cannabis pharmaceutical preparations and the impact on their concentrations after the addition of tocopherol acetate as antioxidant (AOX).

		plain MCT				MCT + AOX				plain preparations in MCT				preparations in MCT+ AOX			
	**BLANK**	**A**	**B**	**C**	**D**	**A**	**B**	**C**	**D**	**A**	**B**	**C**	**D**	**A**	**B**	**C**	**D**
OX 230	nd	nd	nd	nd	nd	nd	nd	nd	nd	-	-	nd	-	-	-	nd	-
OX 270	nd	nd	nd	nd	nd	nd	nd	nd	nd	-	-	nd	-	-	-	nd	-

Nd= not detectable that should be intended as inferior to 0.001%. Preparation methods are summarized as: method **A** Hazekamp, **B** Cannazza, **C** SIFAP and **D** Calvi; see the main text for details.

OX 230= sum of hydroxyl-, hydroperoxyl-, epidioxy- and cheto- oleic (C18:1) fatty acids
OX 270= sum of cheto-linoleic (C18:2) and linolenic (C18:3) fatty acids

Table S7. Percentage of trienes conjugated fatty acids in olive oils and cannabis pharmaceutical preparations and the impact on their concentrations after the addition of tocopherol acetate as antioxidant (AOX).

		plain oils				oils + AOX				plain preparations				preparations + AOX			
	**BLANK**	**A**	**B**	**C**	**D**	**A**	**B**	**C**	**D**	**A**	**B**	**C**	**D**	**A**	**B**	**C**	**D**
C18:3	0.24	0.38	0.42	0.38	0.31	0.31	0.38	0.28	0.27	0.5	0.38	0.13	0.3	0.51	0.32	0.3	0.22
c,t t,c C18:2	0.69	0.7	0.92	0.73	0.89	0.82	0.83	0.79	0.81	0.92	0.78	0.51	0.69	0.94	0.78	0.76	0.83
t,t C18:2	0.01	0.01	0.01	0.01	0.01	0.02	0.01	0.01	0.01	0.02	0.02	0.01	0.01	0.07	0.02	0.02	0.01

Nd= not detectable that should be intended as inferior to 0.001%. Preparation methods are summarized as: method **A** Hazekamp, **B** Cannazza, **C** SIFAP and **D** Calvi; see the main text for details.

c,t t,c C18:2= cis,trans and trans,cis linoleic acid
t,t C18:2 = trans, trans linoleic acid

Table S8. Percentage of trienes conjugated fatty acids in MCT oils and cannabis pharmaceutical preparations and the impact on their concentrations after the addition of tocopherol acetate as antioxidant (AOX).

		plain MCT				MCT + AOX				plain preparations in MCT				Preparations in MCT + AOX			
	**BLANK**	**A**	**B**	**C**	**D**	**A**	**B**	**C**	**D**	**A**	**B**	**C**	**D**	**A**	**B**	**C**	**D**
C18:3	nd	nd	nd	nd	nd	nd	nd	nd	nd	-	-	nd	-	-	-	nd	-
c,t t,c C18:2	nd	nd	nd	nd	nd	nd	nd	nd	nd	-	-	nd	-	-	-	nd	-
t,t C18:2	nd	nd	nd	nd	nd	nd	nd	nd	nd	-	-	nd	-	-	-	nd	-

Nd= not detectable that should be intended as inferior to 0.001%. Preparation methods are summarized as: method **A** Hazekamp, **B** Cannazza, **C** SIFAP and **D** Calvi; see the main text for details.

c,t t,c C18:2= cis,trans and trans,cis linoleic acid
t,t C18:2 = trans, trans linoleic acid

Table S9. Volatile carbonyl compounds (VCCs) in olive oils and cannabis pharmaceutical preparations and the impact on their concentrations after the addition of tocopherol acetate as antioxidant (AOX).

		plain oils				oils + AOX				plain preparations				preparations + AOX			
	**BLANK**	**A**	**B**	**C**	**D**	**A**	**B**	**C**	**D**	**A**	**B**	**C**	**D**	**A**	**B**	**C**	**D**
Propanal	40.5	46.3	34.3	45.8	43.2	41.3	29.5	40.5	40.0	8.5	4.8	2.4	62.4	7.3	16.2	17.9	53.1
Butanal	20.5	35.1	33.1	28.7	23.2	30.0	27.3	25.6	20.2	16.2	4.0	27.1	33.1	3.6	10.9	5.2	28.5
Pentanal	5.7	8.1	8.5	6.6	6.9	7.9	9.0	7.3	7.3	4.6	6.1	6.1	5.0	4.0	3.2	3.0	5.3
Hexadienal	6.6	6.9	5.2	6.2	5.8	5.7	3.6	6.5	4.9	2.2	3.0	2.2	2.6	1.4	2.4	1.6	5.2
2-Pentanone	21.1	23.2	16.5	20.8	20.0	18.2	13.7	20.8	21.2	2.0	5.9	11.7	6.0	4.0	9.2	4.7	5.8
Hexanal	29.2	34.3	27.9	34.9	27.0	25.5	20.9	29.0	14.4	4.7	2.7	1.7	4.4	4.6	6.7	5.6	16.5
Hexane	66.8	67.5	86.6	63.7	62.8	76.0	98.8	63.9	73.4	51.1	46.1	130.3	71.2	64.2	53.1	57.7	66.7
Hexanone	2.1	2.1	1.7	2.2	1.6	1.7	1.5	1.9	1.6	0.7	0.8	1.1	1.6	0.3	1.0	1.6	1.5
Heptenal	3.6	3.8	5.6	3.9	3.0	3.0	4.0	2.6	2.3	6.8	21.7	12.9	16.8	1.8	4.3	4.3	2.1
Heptanal	9.1	10.2	12.3	9.7	10.9	11.9	16.0	8.9	12.4	3.0	12.9	16.7	10.4	14.9	7.0	6.1	10.6
Octenone	2.0	2.0	1.8	2.2	2.2	1.7	1.6	1.5	2.0	3.6	2.2	6.2	7.6	2.8	4.5	7.2	6.9
2-Octenal	1.2	1.3	2.0	1.4	1.1	1.4	2.1	1.1	1.3	7.9	6.0	5.4	3.5	3.4	3.0	2.0	7.8
Octanale	10.8	15.6	30.6	14.6	9.3	17.5	35.1	13.0	11.4	9.5	10.5	12.8	8.5	9.0	9.4	8.1	7.9
Nonenal	34.0	32.1	36.4	33.1	25.9	30.7	35.6	26.5	31.0	22.4	24.3	57.8	24.2	20.6	19.5	26.0	19.8
Nonanal	204.6	214.3	247.4	205.9	234.0	243.4	309.7	188.1	251.1	245.6	317.0	229.0	223.6	234.8	201.8	182.3	181.2
Decenal	1.4	1.9	3.2	1.7	1.9	1.6	2.5	1.5	1.4	1.8	4.9	4.7	5.7	1.6	2.0	4.0	1.8
Decanal	8.6	12.7	25.6	12.0	7.9	17.1	29.9	11.2	9.0	10.3	22.0	14.1	15.7	10.3	13.7	12.5	9.0
Undecanal	7.3	9.9	13.6	8.5	6.0	7.0	11.2	4.7	9.2	1.4	3.7	2.5	2.8	3.2	1.2	3.9	4.2
Tridecanal	2.1	0.4	0.2	0.2	0.3	0.3	0.3	0.3	0.3	0.3	4.6	4.1	3.2	0.7	2.8	1.9	2.2
Tetradecanal	1.7	1.9	1.7	1.3	1.8	1.9	1.9	1.0	1.9	3.6	3.2	1.8	2.5	2.6	2.1	4.8	1.2
Pentadecanal	8.3	10.6	11.4	9.2	10.9	11.9	14.4	9.3	12.7	11.9	13.6	11.3	7.8	12.5	9.9	8.2	9.4

Nd= not detectable that should be intended as inferior to 0.1 mg/kg. Preparation methods are summarized as: method **A** Hazekamp, **B** Cannazza, **C** SIFAP and **D** Calvi; see the main text for details.

Table S10. Volatile carbonyl compounds (VCCs) in MCT oils and cannabis pharmaceutical preparations and the impact on their concentrations after the addition of tocopherol acetate as antioxidant (AOX).

		plain MCT				MCT + AOX				plain preparations in MCT				Preparations in MCT + AOX			
	**BLANK**	**A**	**B**	**C**	**D**	**A**	**B**	**C**	**D**	**A**	**B**	**C**	**D**	**A**	**B**	**C**	**D**
Propanal	nd	nd	nd	nd	nd	nd	nd	nd	nd	-	-	nd	-	-	-	nd	-
Butanal	nd	nd	nd	nd	nd	nd	nd	nd	nd	-	-	nd	-	-	-	nd	-
Pentanal	nd	nd	nd	nd	nd	nd	nd	nd	nd	-	-	nd	-	-	-	nd	-
Hexadienal	nd	nd	nd	nd	nd	nd	nd	nd	nd	-	-	nd	-	-	-	nd	-
2-Pentanone	nd	nd	nd	nd	nd	nd	nd	nd	nd	-	-	nd	-	-	-	nd	-
Hexanal	nd	nd	nd	nd	nd	nd	nd	nd	nd	-	-	nd	-	-	-	nd	-
Hexane	nd	nd	nd	nd	nd	nd	nd	nd	nd	-	-	nd	-	-	-	nd	-
Hexanone	nd	nd	nd	nd	nd	nd	nd	nd	nd	-	-	nd	-	-	-	nd	-
Heptenal	nd	nd	nd	nd	nd	nd	nd	nd	nd	-	-	nd	-	-	-	nd	-
Heptanal	6.8	8.2	6.9	6.9	7.3	8.0	6.5	8.0	7.0	-	-	5.1	-	-	-	4.9	-
Octenone	nd	nd	nd	nd	nd	nd	nd	nd	nd	-	-	nd	-	-	-	nd	-
2-Octenal	nd	nd	nd	nd	nd	nd	nd	nd	nd	-	-	nd	-	-	-	nd	-
Octanale	120.3	89.7	117.2	86.4	115.7	86.1	110.1	89.1	113.8	-	-	108.5	-	-	-	111	-
Nonenal	nd	nd	nd	nd	nd	nd	nd	nd	nd	-	-	nd	-	-	-	nd	-
Nonanal	6.8	5.6	8.8	3.4	6.9	4.6	5.8	5.7	4.4	-	-	13	-	-	-	7.3	-
Decenal	nd	nd	nd	nd	nd	nd	nd	nd	nd	-	-	nd	-	-	-	nd	-
Decanal	46.9	36.2	46.3	34.8	45.9	34.5	43.7	35.7	44.9	-	-	77.3	-	-	-	50.4	-
Undecanal	nd	nd	nd	nd	nd	nd	nd	nd	nd	-	-	nd	-	-	-	nd	-
Tridecanal	nd	nd	nd	nd	nd	nd	nd	nd	nd	-	-	nd	-	-	-	nd	-
Tetradecanal	nd	nd	nd	nd	nd	nd	nd	nd	nd	-	-	nd	-	-	-	nd	-
Pentadecanal	nd	nd	nd	nd	nd	nd	nd	nd	nd	-	-	nd	-	-	-	nd	-

Nd= not detectable that should be intended as inferior to 0.1 mg/kg. Preparation methods are summarized as: method **A** Hazekamp, **B** Cannazza, **C** SIFAP and **D** Calvi; see the main text for details.
